# Implementation of an Online Mental Health Website for the Early Intervention in Psychosis Services, Developed for the Early Youth Engagement (EYE‐2) Trial: A Cross‐Sectional Survey Study of Clinical Barriers and Facilitators to Normalisation

**DOI:** 10.1111/eip.70055

**Published:** 2025-05-29

**Authors:** Elizabeth Robson, Kathryn Greenwood

**Affiliations:** ^1^ Department of Psychology University of Sussex Falmer UK; ^2^ Department of Research and Development Sussex Partnership NHS Foundation Trust Hove UK

**Keywords:** digital, early intervention, implementation, psychosis, schizophrenia

## Abstract

**Introduction:**

Disengagement is a problem for early intervention in psychosis (EIP) services. Access to trusted information on a website might help to overcome some of the problems associated with disengagement. Clinician and organisational engagement are integral to the implementation and uptake of online resources.

**Aims and Objectives:**

A theory‐driven approach used the normalisation process theory (NPT) to investigate the implementation of an NHS psychoeducational website developed for the Early Youth Engagement Project (EYE‐2). The aim was to establish barriers and facilitators to website use.

**Methods:**

A cross‐sectional survey study was used; 36 EIP clinicians in Sussex were asked about their attitudes towards introducing the website and using it in appointments. Accessibility, usability and internet skills were also measured.

**Results:**

A key implementation barrier was lack of familiarity with the website and its content, which inhibited use. Poorer scores in the NPT ‘Collective Action’ construct relating to Skillset Workability and Relational Integration (staff confidence and ability) suggested that clinicians lacked confidence in their skills and ability to introduce the website in clinical sessions. Findings suggest that clinicians, might have lower operational skills compared to the general population.

**Conclusion:**

Embedding of nominated digital leads in teams as well as appropriate training is required to promote familiarity, confidence and enhance digital skills. Larger studies are required to establish the replicability of our findings.

## 1 Introduction

Poor engagement or disengagement before the end of the recommended treatment period is a barrier to services that provide early intervention for people with psychosis (EIP) (Doyle et al. 2014; Robson and Greenwood 2022). Reasons reported for non‐engagement include problems attending appointments (Cowan et al. 2020; O'Keeffe et al. 2016; Tindall et al. 2018, 2020); discomfort around interacting with mental health professionals and coming to NHS buildings (Tindall et al. 2020; Hansen et al. 2018; O'Keeffe et al. 2016). Remote access to trusted information about psychosis and treatment options might help overcome some of these barriers and maintain engagement for longer (Robson and Greenwood 2022). Recent research has found that service users in psychosis care (Aref‐Adib et al. 2019; Eisner et al. 2023) and first episode psychosis (FEP) services (Abdel‐Baki et al. 2017; Lal et al. 2018, 2020) are in favour of receiving mental health care from lead practitioners via digital devices, and previous studies have found online stand‐alone psychoeducational models (i.e., informational resources without a psychotherapeutic element such as CBT) are feasible, acceptable and effective for people with psychosis (Laine et al. 2019; Rotondi et al. 2010).

However, the implementation of digital interventions into routine practice is challenging (Greenhalgh and Abimbola 2019; Torous et al. 2021), and one key component is the perceptions and attitudes of clinical staff (Ajzen 2012; Finch et al. 2018; Corrigan et al. 2001).

Although studies that focus on clinical implementation of digital interventions in psychosis services are rare, findings suggest that practitioners are positive about delivering digital healthcare (Allan et al. 2020). However, concerns have been raised about the lack of resources, staff time pressures and financial limitations impacting wider implementation (Allan et al. 2020).

The current body of literature is small and lacks theoretical grounding; a recent scoping review looked at theory‐driven implementation studies of digital health interventions for people with psychosis and bipolar (Ball et al. 2025). Twelve studies were identified across a range of digital mental health interventions, four of which used an implementation framework; three of those utilised the normalisation process theory (NPT).

The NPT (May et al. 2009; May and Finch 2009) focuses on the role of healthcare organisations and clinicians in providing scaffolding to help understand how new practices become routine through interactive goal‐directed actions that introduce new or modified ways of working. The NPT identifies four core psychological constructs, each with four sub‐constructs: (i) Coherence relates to understanding the value of the intervention and how it is different from current practices; (ii) Cognitive Participation refers to elements of engagement; (iii) Collective Action incorporates organisational support, resources and staff confidence in adoption; and (iv) Reflexive Monitoring is the process of appraisal, modification and user/organisational feedback. The framework is summarised in detail in Table 1.

**TABLE 1:** Summary of the NPT constructs and sub‐constructs for normalisation mechanisms.

NPT construct	Sub‐constructs	Definition
Coherence: Individual and collective sense‐making	Differentiation	Understanding how the intervention differs from what happens already
	Communal specification	Understanding what this means for immediate team working practices and in relation to other organisational teams
	Individual specification	Understanding how the intervention affects individual working practices
	Internalisation	Understanding of the worthwhileness of the intervention on individual working practices
Cognitive participation: Engagement	Initiation	Knowing that there are key people (i.e., managers) who are involved in driving the intervention forward
	Legitimisation	Believing the intervention is right and that individuals can make a valuable contribution through their work
	Enrolment	Being open to adopting new practices with colleagues to implement the intervention
	Activation	Invested in continuing with actions required to support the intervention in everyday practice
Collective action: The activities involved to make the new practices happen	Interactional workability	The work required with objects and practices that integrate the intervention into working practice
	Relational integration	The confidence and ability of staff to integrate the intervention and maintain the stability of working relationships
	Skill set workability	Work is allocated to staff that have the skills to deliver the intervention
	Contextual integration	The organisation provides the appropriate resources and support to deliver the intervention and deal with challenges
Reflexive monitoring: Appraisal of the benefits of the intervention	Systemisation	The organisation provides feedback to appraise the success of the intervention
	Communal appraisal	Staff evaluation of the worthwhileness of the intervention
	Individual appraisal	Individual evaluation of the worthwhileness of the intervention
	Reconfiguration	Individual and collective appraisal of the intervention to inform areas where modification is required

In this study, the NPT framework was used to evaluate the implementation of a new online psychoeducational resource for EIP services developed as part of the Early Youth Engagement Project (EYE‐2) (Greenwood et al. 2021, 2023). As gatekeepers of this resource, it is important that EIP practitioners have an awareness and understanding of the website and can use it in routine practice. No previous study has used a theory‐driven approach to evaluate a web‐based psychoeducational resource for people with serious mental health problems. Findings from a similar study that evaluated a resource for carers of people with psychosis and bipolar (Lobban et al. 2020) found.

Constructs from the NPT were evaluated, supplemented with measures relating to user experience, clinician digital skills and organisational accessibility (i.e., Wi‐Fi and device access) to understand barriers and facilitators for clinicians using the website. The Collective Action, sub‐construct *interactional workability* incorporates user experience, a major and well‐established science concerned with behaviours and motivations that drive people to interact with a digital product (Sauro 2015). A key component of the *Skillset Workability* sub‐construct is clinician knowledge and skills, which have previously been identified as a barrier to digital implementation in mental health services (Corrigan et al. 2001; Cliffe et al. 2020). Finally, barriers around access to suitable devices and adequate Wi‐Fi connection (Aref‐Adib et al. 2019; Camacho and Torous 2022) can sit in the *Contextual Integration* sub‐construct.

The hypothesis that older age would be associated with poorer internet skills was also tested.

## 2 Methods

### 2.1 Research Design

A mixed methods cross‐sectional survey design with data collected at three points during a website training session.

### 2.2 Participants, Clinical Context and Setting

Sussex EIP services participated in the original EYE pilot study in 2013 (Greenwood et al. 2025) and the EYE‐2 training, independent of the main EYE‐2 study in 2018 (Greenwood et al. 2021, 2023). All six EIP teams (approximately 90 clinicians) were invited to attend a follow‐up website training session from May to July 2022 delivered online due to the Coronavirus pandemic.

### 2.3 The Likemind Website

**FIGURE 1:**
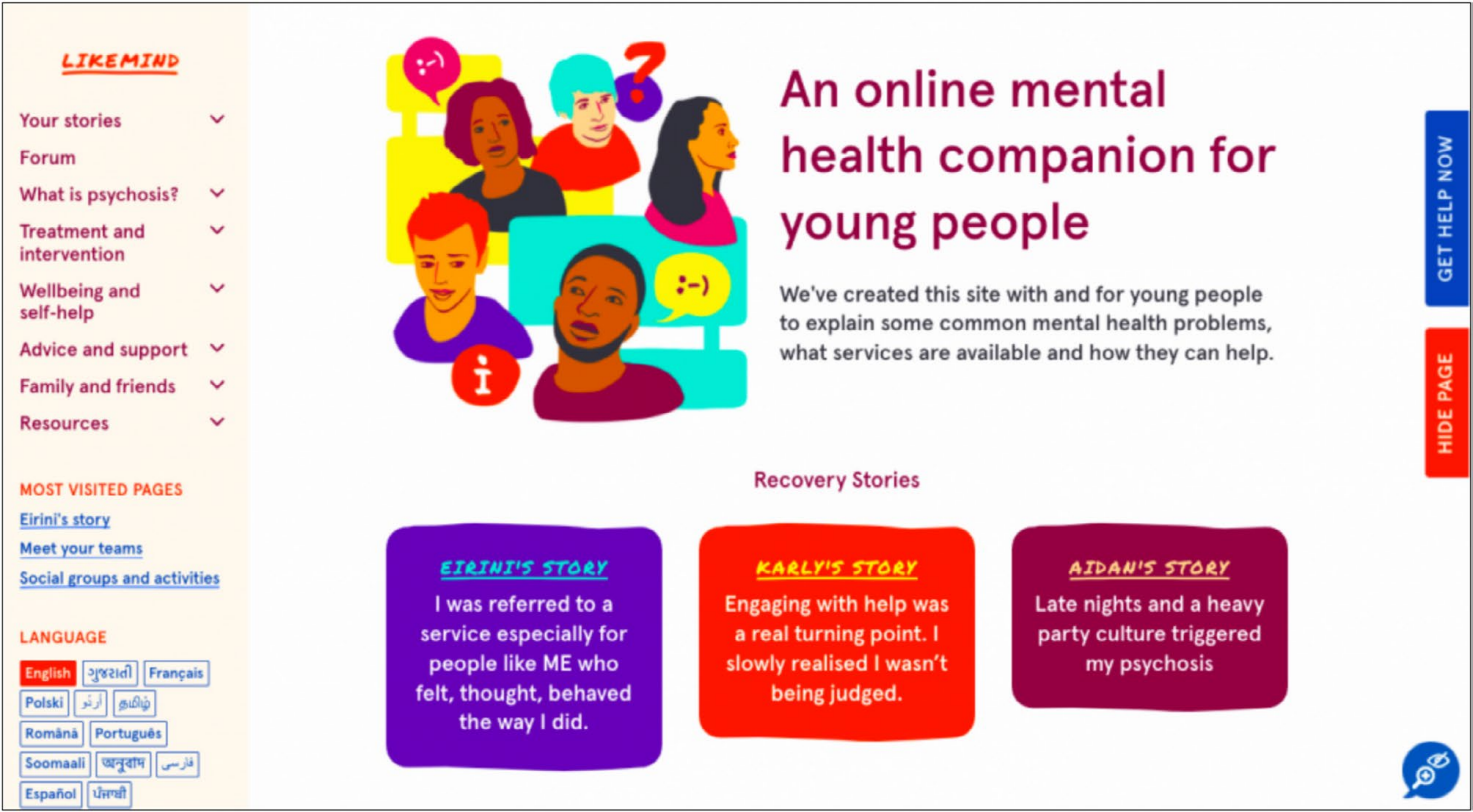
The Likemind homepage.

The EYE‐2 intervention is a new motivational youth engagement approach to EIP care, aimed at improving engagement outcomes for 14‐ to 35‐year‐olds with FEP. The intervention includes the information provided in booklets and as a psychoeducational website. The Likemind website (National Health Service 2019) provides a trusted, up‐to‐date, easily accessible resource for clinicians and service users. It contains information about psychosis, EIP services and treatments, real stories from people who experience psychosis, as well as advice about well‐being, self‐help, recovery and substance use. There is an interactive forum and resource section for service users, family and friends, and clinicians containing useful contacts, leaflets and videos. Figure 1 shows the website homepage.

The website was developed by William Joseph, a certified B Corporation (part of a non‐profit network and global movement for businesses to provide benefits for people, communities, and the planet). They create accessible products, services, and brands for charities, the National Health Service (NHS), and academic institutions working for positive change. Feedback from around 40 stakeholders, including researchers, service users, friends, family, EIP clinicians and NHS digital specialists, helped to establish the optimum design across several iterations. The website is designed to be accessible to people experiencing cognitive difficulties, and content can be translated into multiple languages.

### 2.4 Instruments and Assessments

#### 2.4.1 Preliminary Questionnaire

To measure usability, the 20‐item Internet Skills Questionnaire (ISS) (Grošelj et al. 2020) was used, which has good internal consistency (*α* = 0.74–0.92) (Grošelj et al. 2020) and has been validated in large‐scale general population samples (Grošelj et al. 2020; van Deursen et al. 2016). Mean scores were calculated for the total score and each sub‐scale (operational skills, navigation information skills, social skills and creative skills).

To measure organisational accessibility, staff gave ratings on a 5‐point Likert scale for Wi‐Fi accessibility while at work (*None to Excellent*) and how likely they would be to access the internet during clinical sessions with a computer, laptop, tablet or smartphone (*very unlikely to very likely*). To measure how much the website was being used in clinical teams, we asked them to report how many service users on their caseload aged 14–35 they had used the website with or recommended it to in the last 6 months. Measurement was in quartiles (0%, 1%–25%, 26%–50%, 51%–75% and 76%–100%), where 0% was recorded; respondents were asked if they knew about the website or not.

#### 2.4.2 Mid‐Training Questionnaire

After an introduction to the website and time for familiarisation, we measured the clinician's user experience with the validated 8‐item Standardised User Experience Percentile Rank Questionnaire (SUPR‐Q) (Sauro 2015). The scale has high internal consistency (*α* = 0.86) (Sauro 2015), is widely used commercially (Sauro 2015, 2018) and has been used to evaluate mental‐health‐focused websites (Hartmann et al. 2023; Dubov et al. 2021; Merkouris et al. 2022). Items 3, 4 and 5 were re‐worded to reflect the nature of the Likemind website, replacing ‘purchasing’ with ‘getting information’, ‘conducting business’ with ‘using the website with service users and families’ and ‘friend, family member or carer of someone with psychosis’ rather than ‘friend or colleague’.

#### 2.4.3 Post‐Training Questionnaire

To measure implementation constructs, the validated Normalisation Measure Development Assessment Tool (NoMAD) (Finch et al. 2018) was used to collect clinician opinions and attitudes across the NPT constructs as well as demographic and clinical information (May et al. 2009; May and Finch 2009). It also captured ratings for familiarity, feelings about current practice and potential for future practice. NoMAD shows high internal consistency (*α* = 0.89) (Finch et al. 2018) and has been used as an evaluation tool for the EYE‐2 project (Greenwood et al. 2021; May et al. 2022).

#### 2.4.4 Qualitative Data

Free text boxes in the pre‐ and post‐training questionnaires asked participants to provide feedback on their perspectives and preferences regarding electronic or paper versions of the resources (the Likemind booklet resources) and anything that prevented website use or made it harder, as well as general feedback.

### 2.5 Procedure

After informed consent was obtained, attendees were invited to complete the survey questions at the specified time points; NoMAD questions were collected from lead practitioners only.

### 2.6 Analysis and Missing Data

Three missing data points from one participant in the NoMAD scale were substituted with their average score for that construct.

Data was analysed and visualised using R‐Studio. To see how clinician internet skills compared against the general population, a one‐sample *Z*‐test was used to evaluate scores from the three relevant internet skills sub‐scales against a large general population study (Büchi et al. 2017). Pearson correlation evaluated associations between age and internet skills, testing the hypothesis that older age is associated with poorer digital skills. A priori power analysis suggested that at least 50 participants will generate sufficient statistical power of 0.8 to detect a moderate effect size of 0.4 with alpha set at 0.05.

Qualitative data were extracted from three free text boxes that asked: (i) whether clinicians favoured a specific format for the resources (paper or electronic), (ii) if there were any barriers for those who had not used the resources or only used them minimally, and (iii) feedback about the website in general. Deductive thematic analysis was conducted and mapped onto the NPT constructs; coding and mapping were reviewed by the second author to reach consensus on the final theme structure.

## 3 Results

### 3.1 Demographics

In total, 36 clinicians consented; the demographic characteristics of respondents are summarised in Table 2.

**TABLE 2:** Demographic and clinical variables (*n* = 36).

Categorical variables		*n* (%)
Gender	Female	29 (80.6)
	Male	6 (16.7)
	Declined	1 (2.8)
Ethnicity	White	34 (94.4)
	Other	1 (2.8)
	Declined	1 (2.8)
Team	Team 1	8 (22.2)
	Team 2	5 (13.9)
	Team 3	4 (11.1)
	Team 4	7 (19.4)
	Team 5	6 (16.7)
	Team 6	6 (16.7)
Job role	Mental Health Nurse	12 (33.3)
	Clinical Psychologist	6 (16.7)
	Occupational therapist	6 (16.7)
	Social Worker	4 (11.1)
	Support Worker	3 (8.3)
	Trainee Clinical Psychologist	1 (2.8)
	Psychiatrist	1 (2.8)
	Declined	3 (8.3)
LP	Yes	23 (63.9)
	No	12 (33.3)
	Declined	1 (2.8)

Continuous variable	*M* (SD)	*n*
Age	43.0 (8.5)	33

### 3.2 Use of the Likemind Website

In order to evaluate how the website was being used in EIP teams in Sussex, 33 clinicians provided data about website use (summarised in Table 3). This included looking at the website in clinical sessions, sending a web link, writing down the web link for service users, or printing out information from the web pages. Fifteen (45.4%) had used the website, 10 of whom were lead practitioners. Of the 18 clinicians who said they had not used the website, half did not know about it, 5 of those had been in EIP services less than 2 years and four between 2 and 5 years. Most clinicians had recommended or used the website with less than 50% of their caseload.

**TABLE 3:** Clinician website use with service users 14–35 years old.

Frequency of use or recommendation	Total (*n* = 33)	LP's (*n* = 21)
% of service users with whom the website was used or recommended	*n* (% of samples)	*n* (% of LPs)
1%–25% of service users	7 (21)	4 (19)
26%–50% of service users	6 (18)	5 (24)
51%–75% of service users	2 (6)	1 (5)
76%–100% of service users	0 (0)	0 (0)
None—I did not know about it	9 (27)	6 (27)
None—I know but have not used it	9 (27)	5 (24)

Only three clinicians shared the website during clinical sessions; two of those were lead practitioners.

### 3.3 NoMAD Ratings for Familiarity, Current Practice and Potential for Normal Practice (Score Range 0–10)

Using the website in clinical sessions did not feel familiar to lead practitioners (*Median* 1, *Range* 0–8, *n* = 15) nor did it feel like it was current practice (*Median* 1, *Range* 0–6, *n* = 15). Most believed that the practice of sharing the website had the potential to become routine (*Median* 8, *Range* 5–10, *n* = 15), suggesting confidence in the value of this approach; see (Figures S1–S3 for frequency distributions).

### 3.4 Evaluation of Normalisation Mechanisms, Barriers and Facilitators of Clinician Website Use Using the NPT Framework

Barriers and facilitators were arranged according to the NPT constructs. Fifteen of the 23 lead practitioners (65%) completed the NoMAD measurement scale.

**FIGURE 2:**
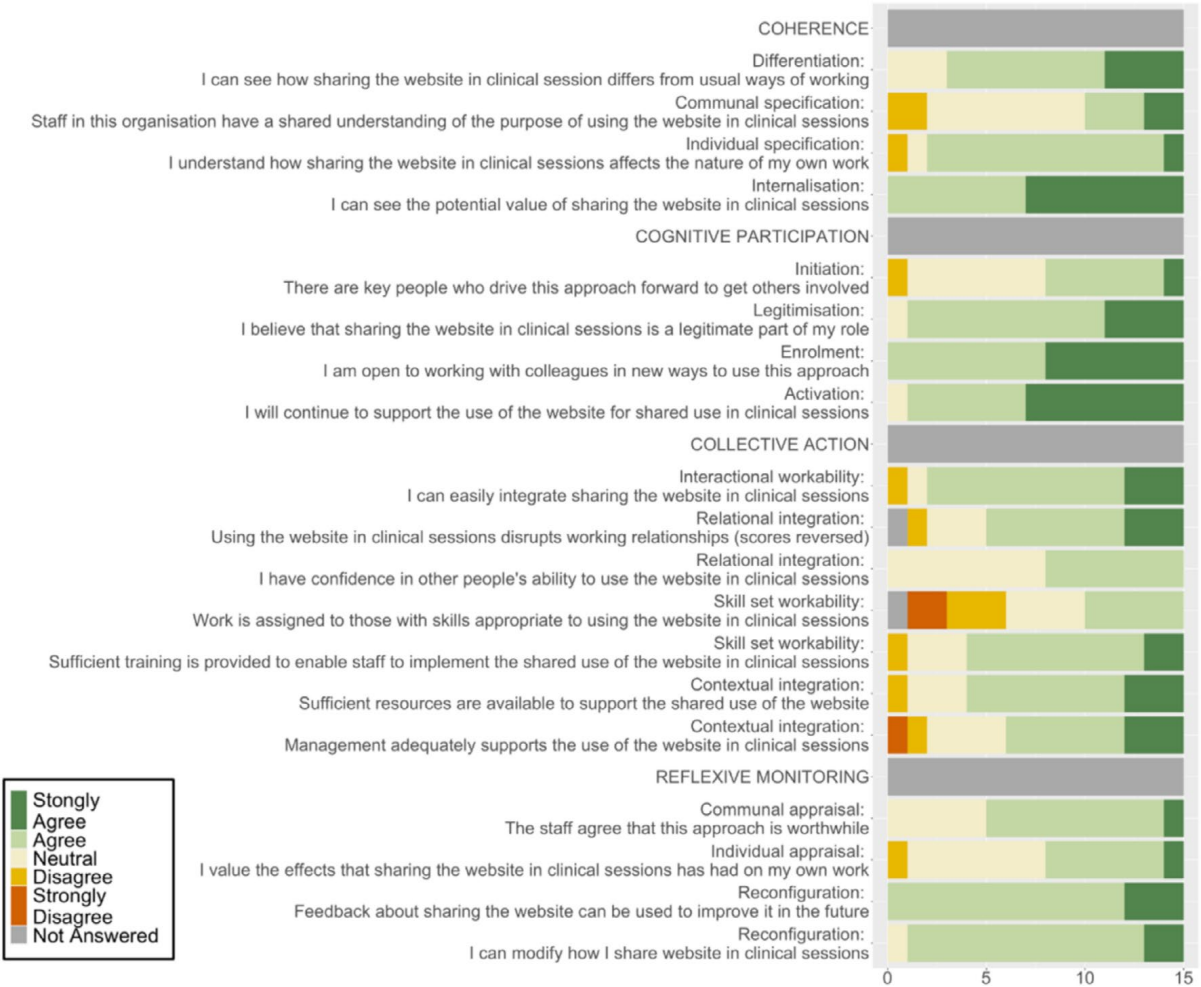
Frequency histogram showing ratings for individual NoMAD items.

Overall, lead practitioner attitudes were positive for all constructs. Clinician ratings for each NoMAD item are illustrated in Figure 2; frequency distributions for individual average scores across each core construct can be found in Figures S4–S7.

### 3.5 Evaluation of NPT Construct ‘Coherence’ for Normalisation Mechanisms

#### 3.5.1 Coherence: NoMAD Scores (Score Range 1 Strongly Agree to 5 Strongly Disagree)

Coherence was the second most positively scored construct (Median 2.00/agree, Range 1.25–3.00), suggesting that the potential value of sharing the website with service users was understood by lead practitioners. For the poorest scoring sub‐construct, *Communal Specification* (see Figure 2), most opinions were neutral, reflecting a lack of shared understanding about the purpose of the website use in clinical sessions.

#### 3.5.2 Coherence: Qualitative Data

Despite positive scores in the NoMAD scale, qualitatively, specific barriers to website use related to a lack of understanding about how the use of the website differed from usual work (*Differentiation*) or had potential value (*Internalisation*). A core theme was clinicians expressing that the website was not preferred; a large sub‐theme was that clinicians favoured the paper booklets; one clinician said that they used their own resources, another only signposted in certain circumstances.

I find it more convenient [using the booklets]. I like to have physical resources (Mental Health Nurse).

I signpost to online when paper [is] not available, during phone/online contact, for people who show preference for online stuff (Occupational Therapist).

I have used my own materials. Likemind wasn't being widely used in the team when I arrived a year ago (Clinical Psychologist).

This suggests a lack of understanding of the benefits of using online resources vs. paper copies, such as the benefits of the language translation feature. However, a few identified this as beneficial.

…if English is a second language, then they can have time to digest the information in written format (Occupational Therapist).

### 3.6 Evaluation of NPT Construct ‘Cognitive Participation’ for Normalisation Mechanisms

#### 3.6.1 Cognitive Participation: NoMAD Scores (Score Range 1 Strongly Agree to 5 Strongly Disagree)

This was the most positively scored construct; on average, most people agreed or strongly agreed with the NoMAD questions (Median 1.75, Range 1.00–2.75), suggesting that they viewed it as part of their role at work and were open to using the website. The poorest scoring sub‐construct was *Initiation* (see Figure 2) suggesting uncertainty about who was taking responsibility for promoting the intervention.

#### 3.6.2 Cognitive Participation: Qualitative Data

One barrier identified was around the *legitimisation* sub‐construct, where a large theme was that the youth focus wasn't sufficiently inclusive for clinicians' older EIP service users.

It would be good for the information to be more inclusive for over 35s so all service users can benefit and not feel excluded (Mental Health Nurse).

### 3.7 Evaluation of NPT Construct ‘Collective Action’ for Normalisation Mechanisms

#### 3.7.1 Collective Action: NoMAD Scores (Score Range 1 Strongly Agree to 5 Strongly Disagree)

Although scores still reflected an overall positive attitude, Collective Action was the poorest scoring NoMAD construct (Median 2.28, Range 1.37–3.29).

Five people disagreed or strongly disagreed with the statement ‘Work is assigned to those with appropriate skills to deliver the intervention’. In the *Skill Set Workability* sub‐construct, the median score was 3/neutral, with a range of 2–5 (see Figure 2). Most respondents rated ‘neutral’ for the *Relational Integration* sub‐construct with a range of 2–3, which suggests a possible lack of confidence in staff skills.

#### 3.7.2 Collective Action: Supplementary Measures Used to Evaluate the Collective Action Sub‐Constructs

##### 3.7.2.1 Interactional Workability: Website User Experience

Website user experience informs the *Interactional Workability* sub‐construct, the actions that help integrate the intervention into working practice. Scores were excellent; most respondents ‘agreed’ (4) or ‘strongly agreed’ (5) with all questions. The total median score was 4.50 (range 1.88–5.00).

##### 3.7.2.2 Contextual Integration: Organisational Resources and Support

Accessibility of Wi‐Fi and devices was evaluated to inform the *Contextual Integration* sub‐construct (organisational resources and support). Findings suggest that this was not a barrier; 97% of respondents rated Wi‐Fi access as Good or Excellent at their team base. Nearly two‐thirds (64%) said the same for access in clinical sessions (e.g., if lead practitioners see service users at their own homes), with a further 20% rating access as Moderate.

Most clinicians also reported having access to a suitable device to use the internet at work and during clinical sessions; the most likely being laptops (82% said it was likely or very likely) or smartphones (76% said it was likely or very likely).

##### 3.7.2.3 Skillset Workability: Internet Skills

To help inform the *Skillset Workability* sub‐construct within Collective Action, we measured clinicians' internet skills on the ISS scale and compared them against a large general population study of internet users aged 14–84 (*M* = 44.4, SD = 17.6, *n* = 970) (Büchi et al. 2017) (see Table 4). Scores suggested that clinicians demonstrated stronger navigational skills but might be lacking in operational skills, integral to some of the processes involved in using online resources (e.g., downloading and saving files). However, our sample is considerably smaller than the size recommended for adequate statistical power.

**TABLE 4:** Scores for internet skills with z‐test comparison.

Internet skills (*n* = 33)	*M* (SD)	Swiss general population (*n* = 970)	*Z*‐test, *p*, 95% CI
		*M* (SD)	
		(Büchi et al. 2017)	
Total score	3.56 (0.61)	Not published	—
Operational skills sub‐scale For example, *I know how to open downloaded files*	4.09 (0.92)	4.46 (1.08)	−1.96, 0.050* 3.72–4.46
Information Navigation sub‐scale For example, *I find it hard to find a website I visited before* (reverse scored)	4.30 (0.68)	3.87 (1.10)	2.26, 0.024* 3.93–4.68
Social sub‐scale For example, *I know which information I should and shouldn't share online*	3.98 (0.92)	3.38 (2.24)	1.53, 0.127 3.21–4.74

*Note:* Scores are on a 5‐point scale where 1 (*not at all true of me*) to 5 (*very true of me*) with items on the Information Navigation sub‐scale are reverse scored. Scores for Creativity sub‐scale have been omitted as they are not considered relevant to website use.

* *p* < 0.05.

Analysis found lower age to be associated with better ‘operational skills’ (*r* = −0.43, *p* = 0.019); again, statistical power was low due to the small sample size.

#### 3.7.3 Collective Action: Qualitative Data

**FIGURE 3:**
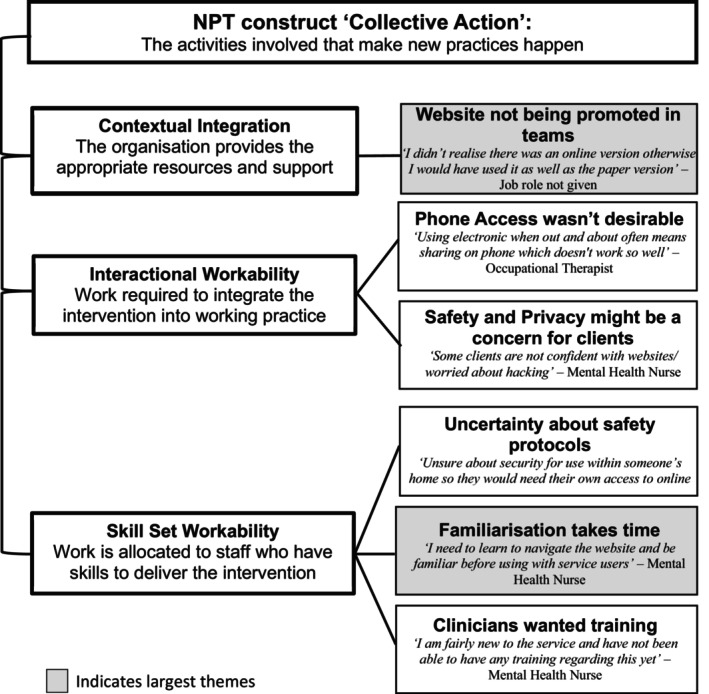
Barriers related to NPT Collective Action sub‐constructs: Themes identified from qualitative feedback.

Collective Action was the construct where clinicians identified the most barriers and suggested that although the website was easy to use, problems relating to lack of training, lack of awareness, and lack of clarity around safety protocols were all barriers to use. The largest themes related to barriers around familiarity. Qualitative data for the collective action construct are summarised in Figure 3. One facilitator identified that the website was easy to use and had clear information.

### 3.8 Evaluation of NPT Construct ‘Reflexive Monitoring’ for Normalisation Mechanisms

#### 3.8.1 Reflexive Monitoring: NoMAD Scores (Score Range 1 Strongly Agree to 5 Strongly Disagree)

Reflexive Monitoring was the second most positively scored construct (alongside Coherence) with a median score of 2.00/agree (range 1.50–2.75), suggesting high confidence in the impact and potential for implementation.

#### 3.8.2 Reflexive Monitoring: Qualitative Feedback

No free text feedback related to reflexive monitoring.

## 4 Discussion

The NPT was used to evaluate the normalisation of the Likemind website use and recommendation for clinicians. Of the nine (55%) who had not used the website, half, did not know about it. Attitudes towards the website and its potential were favourable, but unfamiliarity was a key barrier. In the Coherence construct, clinicians said they preferred existing practices (i.e., using paper versions of the information). In the Cognitive Participation construct, barriers were present due to the youth's focus on the materials. In the Reflexive Monitoring construct, clinicians showed confidence in the potential of the website and its adaptability for future use. Although still relatively positive, the least favourable scores in the NoMAD measure related to Collective Action around skills and ability, suggesting a need for more training and support around digital technologies in clinical settings.

From the ISS, lower scores were reported for operational skills (e.g., downloading and saving files or bookmarking pages), which might be associated with older age. This finding is in line with existing literature from a Dutch general population sample (*n* = 109) (van Deursen and van Dijk 2011) that found older participants had significantly worse operational skills. Other technological‐related variables did not present any barriers: website usability scores were excellent as were Wi‐Fi accessibility and access to suitable devices.

Fifteen of the 33 clinicians (45%) had used the Likemind website in some capacity, but only 3 had shared it with service users in clinical sessions. Most said they recommended or used the resources with less than 50% of service users. Several respondents commented in feedback that they had not used the Likemind resources because of the youth focus of the EYE‐2 (originally aimed at 14–35‐year‐olds). Due to recent policy changes, roughly a third of service users in Sussex EIP services are now over 35. Some adjustments have already been made to the resources to reflect this change.

Lack of familiarity is a problem cited in other digital implementation literature in psychosis (Aref‐Adib et al. 2019) and EIP services (Allan et al. 2019). The lack of website promotion in teams meant that nine clinicians (27%), mostly newer staff, did not know about it. Across the four core NPT constructs, scores were generally positive; however, qualitatively, clinicians preferred to use paper booklets, which suggests a lack of understanding of how the website differs from usual ways of working (coherence). Namely, how it can be accessed remotely, without the need for printing or distribution, and can easily be translated into multiple languages (although this feature was mentioned by some respondents).

Our findings are echoed in the larger EYE‐2 trial, where preliminary results suggested that the website was not used as much as anticipated, and this study gives more detailed insight as to why this might be the case.

In the Cognitive Participation construct, staff were uncertain about who was responsible for driving the website promotion (the *Initiation* sub‐construct), which could explain the lack of awareness in teams and for new staff members.

In the Collective Action construct, staff skills and confidence (*Skillset Workability*) were a barrier, which has also been cited in previous research. For example, in a survey study of clinicians working in CAMHS (*n* = 154), 42% expressed feeling unskilled or underconfident about technologies in the arena of child mental health care (Cliffe et al. 2020). In psychosis care, deficits in staff skills have often been identified as a barrier to digital implementation, and slower completion of tasks related to this has been cited as adding to clinical time pressures (Aref‐Adib et al. 2019). Possible lower operational skills might be a contributing factor and are in line with previous findings (Büchi et al. 2017), which were also moderately associated with older age. It could be that the practical nature of clinical work gives less exposure to the sort of operational skills that might be valuable for using websites, particularly for older clinicians who may not have developed digital skills during core professional training in the same way that younger people might. These findings suggest a need for a digital lead in each team to promote the website and coordinate training and induction for new staff.

In the Collective Action sub‐construct *Interactional Workability*, all but two lead practitioners agreed or strongly agreed that it would be possible to integrate website use into their existing work. This is supported by excellent website usability scores and qualitative feedback that mentions clear and easily accessible information.

Reflexive monitoring scores suggest high confidence in the ability to adapt and modify the intervention to fit into clinical work; clinicians also rated highly the ‘potential for future practice’ (Median 8, Range 5–10). These results suggest that the website is highly regarded suggesting promise for implementation.

The Likemind website is the first of its kind in EIP services; there are two published studies in the psychosis literature that evaluate a psychoeducational website (Laine et al. 2019; Rotondi et al. 2010); however, neither evaluated clinical implementation beyond establishing acceptability and feasibility. In broader research, an evaluation of an online psychoeducational tool in forensic services found that staff attitudes towards the potential of the intervention were good, but that implementation was poor, which they attributed to lack of organisational support. In line with our findings, low general awareness was also a contributing factor (Kip et al. 2020).

### 4.1 Clinical Implications

Favourable clinician attitudes and excellent usability suggest that the use of the Likemind website to support clinical sessions has promise to become normalised practice. However, the lack of skills, awareness, and familiarisation as well as the perceived lack of skills are key barriers to website use.

To address this, it is important that website promotion and routine staff training are employed to help aid normalisation. A nominated digital lead could help coordinate training and website induction for new starters as well as giving staff a main point of contact for queries and feedback. Kip et al. (2020) suggested making digital mental health interventions a permanent fixture on the agenda at team meetings and peer coaching to encourage communication between colleagues. These strategies could help remind colleagues about website use and increase familiarity as well as help highlight the unique benefits and how they can enhance clinical practices. Additionally, there may be a need to enhance clinician operational skills, particularly for older staff members.

Future research should establish more accurately any possible deficits in internet skills for clinical populations compared to the general population. Due to the relatively large number of participants who did not know about the website, it was not meaningful to evaluate correlates of website use; therefore, future research should also aim to identify variables that might predict website use and develop training to increase skills and confidence and ameliorate other barriers.

### 4.2 Limitations

This study was conducted towards the end of a global pandemic resulting in a smaller sample than anticipated; consequently, the findings are largely descriptive. *Z*‐tests and correlation analysis should be interpreted with caution due to low statistical power. The current study was conducted in only one UK NHS trust, which has the potential to limit generalisability. However, key performance indicators for this trust, as reported in the UK National Clinical Audit of Psychosis (NCAP) for Early Intervention care standards (Royal College of Psychiatrists 2022) suggest that the trust is representative of average care standards across the United Kingdom. Globally, although there is some variation, the stand‐alone EIP treatment model employed in this NHS trust follows the original protocol established by the pioneer of this approach, Patrick McGorry and his team, and used internationally (McGorry et al. 1996; McGorry and Killackey 2002). Furthermore, the findings regarding implementation of the online website are consistent with findings from other similar studies (Aref‐Adib et al. 2019; Cliffe et al. 2020; Allan et al. 2019). These consistencies make it likely that the barriers identified will be present in many other EIP settings.

The sample represents around 40% of Sussex EIP staff, possibly those who are most engaged with their job, training and/or innovation, giving opinions that are not entirely representative of the full staff cohort. Finally, due to the nature of survey‐based research, there is also a possibility of response bias.

## 5 Conclusion

The website was found to be highly usable, and lead practitioners gave positive opinions about the potential use of the website as a way of aiding psychoeducation and shared decision‐making in clinical sessions. Key barriers were in collective action around promotion and knowledge of the website and its content as well as a lack of time for familiarisation and a possible deficit in internet operational skills. Future recommendations include a nominated digital lead to promote the website and coordinate regular training and new staff induction. Also, increased priority should be given to digital interventions in team meetings and peer coaching to enhance knowledge, skills and familiarisation. Research should prioritise more robust research methods to establish predictors of website use and replicability of findings.

## Ethics Statement

Ethical approval for the EYE‐2 project (IRAS: 238744) and sub‐study (SA06) was obtained from the London‐Dulwich Research Ethics Committee (REC) (Ref: 18/LO/0362). Informed consent was obtained from all study participants.

## Conflicts of Interest

The authors declare no conflicts of interest.

## Supporting information


**Figure S1.** NoMAD scores for ‘sharing feels familiar’.
**Figure S2.** NoMAD scores for ‘feels like current practice’.
**Figure S3.** NoMAD scores for ‘sharing has potential to become routine practice’.
**Figure S4.** Average scores for coherence (individual and collective sense‐making).
**Figure S5.** Average scores for cognitive participation (clinician engagement).
**Figure S6.** Average scores for collective action (the activities involved to make the new practices happen).
**Figure S7.** Average scores for reflexive monitoring (appraisal of the benefits of the intervention).

## Data Availability

The data that support the findings of this study are available on request from the corresponding author. The data are not publicly available due to privacy or ethical restrictions.

## References

[eip70055-bib-0001] Abdel‐Baki, A. , S. Lal , O. D.‐Charron , E. Stip , and N. Kara . 2017. “Understanding Access and Use of Technology Among Youth With First‐Episode Psychosis to Inform the Development of Technology‐Enabled Therapeutic Interventions.” Early Intervention in Psychiatry 11, no. 1: 72–76. 10.1111/eip.12250. 26011657

[eip70055-bib-0002] Ajzen, I. 2012. “The Theory of Planned Behavior.” In Handbook of Theories of Social Psychology: Volume 1, 438–459. Sage. 10.4135/9781446249215.n22.

[eip70055-bib-0003] Allan, S. , S. Bradstreet , H. McLeod , et al. 2019. “Developing a Hypothetical Implementation Framework of Expectations for Monitoring Early Signs of Psychosis Relapse Using a Mobile App: Qualitative Study.” Journal of Medical Internet Research 21, no. 10: e14366. 10.2196/14366. 31651400 PMC6838692

[eip70055-bib-0004] Allan, S. , H. Mcloed , S. Bradstreet , et al. 2020. “Trial Staff Views on Barriers Recruitment in a Digital Intervention for Psychosis and How to Work Around Them: A Qualitative Study Within a Trial.” Published Online May 13. 10.21203/rs.3.rs-20235/v1. PMC7980120 33666555

[eip70055-bib-0005] Aref‐Adib, G. , T. McCloud , J. Ross , et al. 2019. “Factors Affecting Implementation of Digital Health Interventions for People With Psychosis or Bipolar Disorder and/or Their Family and Friends: A Systematic Review.” Lancet 6, no. 3: 68–70. 10.1016/S2215-0366(18)30302-X 30522979

[eip70055-bib-0006] Ball, H. , E. Eisner , J. Nicholas , P. Wilson , and S. Bucci . 2025. “How Theories, Models, and Frameworks Have Been Used to Implement Digital Health Interventions in Services for People With Severe Mental Health Problems: A Scoping Review.” BMC Public Health 25, no. 1: 1023. 10.1186/s12889-025-22189-2. 40098003 PMC11912717

[eip70055-bib-0007] Büchi, M. , N. Just , and M. Latzer . 2017. “Caring Is Not Enough: The Importance of Internet Skills for Online Privacy Protection.” Information, Communication & Society 20, no. 8: 1261–1278.

[eip70055-bib-0008] Camacho, E. , and J. Torous . 2022. “Introducing an Implementation Framework for Augmenting Care With Digital Technology for Early Psychosis Patients: Theory and Motivation.” Journal of Mental Health 31, no. 6: 816–824. 10.1080/09638237.2021.1922634. 34057008

[eip70055-bib-0009] Cliffe, B. , A. Croker , M. Denne , and P. Stallard . 2020. “Clinicians' Use of and Attitudes Towards Technology to Provide and Support Interventions in Child and Adolescent Mental Health Services.” Child and Adolescent Mental Health 25, no. 2: 95–101. 10.1111/camh.12362. 32307835

[eip70055-bib-0010] Corrigan, P. W. , L. Steiner , S. G. McCracken , B. Blaser , and M. Barr . 2001. “Strategies for Disseminating Evidence‐Based Practices to Staff Who Treat People With Serious Mental Illness.” Psychiatric Services 52, no. 12: 1598–1606. 10.1176/appi.ps.52.12.1598. 11726749

[eip70055-bib-0011] Cowan, T. , M. A. Pope , K. MacDonald , A. Malla , M. Ferrari , and S. N. Iyer . 2020. “Engagement in Specialized Early Intervention Services for Psychosis as an Interplay Between Personal Agency and Critical Structures: A Qualitative Study.” International Journal of Nursing Studies 108: 103583. 10.1016/j.ijnurstu.2020.103583. 32502820

[eip70055-bib-0012] Doyle, R. , N. Turner , F. Fanning , et al. 2014. “First‐Episode Psychosis and Disengagement From Treatment: A Systematic Review.” Psychiatric Services 65, no. 5: 603–611. 10.1176/appi.ps.201200570. 24535333

[eip70055-bib-0013] Dubov, A. , L. Fraenkel , Z. Goldstein , H. Arroyo , D. McKellar , and S. Shoptaw . 2021. “Development of a Smartphone App to Predict and Improve the Rates of Suicidal Ideation Among Transgender Persons (Translife): Qualitative Study.” Journal of Medical Internet Research 23, no. 3: e24023. 10.2196/24023. 33596181 PMC8074983

[eip70055-bib-0014] Eisner, E. , N. Berry , and S. Bucci . 2023. “Digital Tools to Support Mental Health: A Survey Study in Psychosis.” BMC Psychiatry 23, no. 1: 726. 10.1186/s12888-023-05114-y. 37803367 PMC10559432

[eip70055-bib-0015] Finch, T. L. , M. Girling , C. May , et al. 2018. “Improving the Normalization of Complex Interventions: Part 2—Validation of the NoMAD Instrument for Assessing Implementation Work Based on Normalization Process Theory (NPT).” BMC Medical Research Methodology 18, no. 1: 135. 10.1186/s12874-018-0591-x. 30442094 PMC6238372

[eip70055-bib-0016] Greenhalgh, T. , and S. Abimbola . 2019. “The NASSS Framework A Synthesis of Multiple Theories of Technology Implementation.” Studies in Health Technology and Informatics 263: 193–204. 10.3233/SHTI190123. 31411163

[eip70055-bib-0017] Greenwood, K. , R. Chandler , K. Labuschagne , et al. 2025. “The Development and Piloting of an Early Youth‐Engagement (EYE) Model to Improve Engagement of Young People in First Episode Psychosis Services: A Mixed Methods Study.” Early Intervention in Psychiatry 19, no. 1: e13623. 10.1111/eip.13623. 39435971 PMC11730687

[eip70055-bib-0018] Greenwood, K. , C. Jones , N. Yaziji , et al. 2023. “The Early Youth Engagement (EYE‐2) Intervention in First Episode Psychosis: The RCT, Cost‐Effectiveness and Process Evaluation.” British Journal of Psychiatry 226, no. 3: 144–152. 10.1192/bjp.2024.154. 39581220

[eip70055-bib-0019] Greenwood, K. , R. Webb , J. Gu , et al. 2021. “The Early Youth Engagement in First Episode Psychosis (EYE‐2) Study: Pragmatic Cluster Randomised Controlled Trial of Implementation, Effectiveness and Cost‐Effectiveness of a Team‐Based Motivational Engagement Intervention to Improve Engagement.” Trials 22, no. 1: 272. 10.1186/s13063-021-05105-y. 33845856 PMC8042707

[eip70055-bib-0020] Grošelj, D. , A. van Deursen , V. Dolničar , T. Burnik , and A. Petrovčič . 2020. “Measuring Internet Skills in a General Population: A Large‐Scale Validation of the Short Internet Skills Scale in Slovenia.” Information Society 37, no. 2: 63–81. 10.1080/01972243.2020.1862377.

[eip70055-bib-0021] Hansen, H. , S. H. Stige , L. Davidson , C. Moltu , and M. Veseth . 2018. “How Do People Experience Early Intervention Services for Psychosis? A Meta‐Synthesis.” Qualitative Health Research 28, no. 2: 259–272. 10.1177/1049732317735080. 29039239

[eip70055-bib-0022] Hartmann, M. , S. T. Roberts , N. Triplett , et al. 2023. “Development of a Relationship Counselling Website to Identify and Mitigate Risk of Intimate Partner Violence in the Context of Women's PrEP Use.” PLOS Digital Health 2, no. 8: e0000329. 10.1371/journal.pdig.0000329. 37578954 PMC10424861

[eip70055-bib-0023] Kip, H. , F. Sieverink , L. J. E. W. C. van Gemert‐Pijnen , Y. H. A. Bouman , and S. M. Kelders . 2020. “Integrating People, Context, and Technology in the Implementation of a Web‐Based Intervention in Forensic Mental Health Care: Mixed‐Methods Study.” Journal of Medical Internet Research 22, no. 5: 1–24. 10.2196/16906. PMC7284403 32348285

[eip70055-bib-0024] Laine, A. , M. Välimäki , V. Pekurinen , E. Löyttyniemi , M. Marttunen , and M. Anttila . 2019. “Feasibility, Acceptability, and Preliminary Impacts of Web‐Based Patient Education on Patients With Schizophrenia Spectrum Disorder: Quasi‐Experimental Cluster Study.” Journal of Medical Internet Research 21, no. 10: 1–18. 10.2196/13073. PMC6913382 31625952

[eip70055-bib-0025] Lal, S. , A. Abdel‐Baki , S. Sujanani , F. Bourbeau , I. Sahed , and J. Whitehead . 2020. “Perspectives of Young Adults on Receiving Telepsychiatry Services in an Urban Early Intervention Program for First‐Episode Psychosis: A Cross‐Sectional, Descriptive Survey Study.” Frontiers in Psychiatry 11: 1–8. 10.3389/fpsyt.2020.00117. 32194457 PMC7065530

[eip70055-bib-0026] Lal, S. , V. Nguyen , and J. Theriault . 2018. “Seeking Mental Health Information and Support Online: Experiences and Perspectives of Young People Receiving Treatment for First‐Episode Psychosis.” Early Intervention in Psychiatry 12, no. 3: 324–330. 10.1111/eip.12317. 26810026

[eip70055-bib-0027] Lobban, F. , D. Appelbe , V. Appleton , et al. 2020. “IMPlementation of an Online Relatives' Toolkit for Psychosis or Bipolar (IMPART Study): Iterative Multiple Case Study to Identify Key Factors Impacting on Staff Uptake and Use.” BMC Health Services Research 20, no. 1: 219. 10.1186/s12913-020-5002-4. 32183787 PMC7077000

[eip70055-bib-0028] May, C. , and T. Finch . 2009. “Implementing, Embedding, and Integrating Practices: An Outline of Normalization Process Theory.” Sociology 43, no. 3: 535–554. 10.1177/0038038509103208.

[eip70055-bib-0029] May, C. , F. Mair , T. Finch , et al. 2009. “Development of a Theory of Implementation and Integration: Normalization Process Theory.” Implementation Science 4, no. 1: 1–9. 10.1186/1748-5908-4-29. 19460163 PMC2693517

[eip70055-bib-0030] May, C. R. , B. Albers , M. Bracher , et al. 2022. “Translational Framework for Implementation Evaluation and Research: A Normalisation Process Theory Coding Manual for Qualitative Research and Instrument Development.” Implementation Science 17, no. 1: 1–15. 10.1186/s13012-022-01191-x. 35193611 PMC8861599

[eip70055-bib-0031] McGorry, P. D. , J. Edwards , C. Mihalopoulos , S. Harrigan , and H. J. Jackson . 1996. “The Early Psychosis Prevention and Intervention Centre (EPPIC): An Evolving System of Early Detection and Optimal Management.” Schizophrenia Bulletin 22: 305–326. 8782288 10.1093/schbul/22.2.305

[eip70055-bib-0032] McGorry, P. D. , and E. J. Killackey . 2002. “Early Intervention in Psychosis: A New Evidence Based Paradigm.” Epidemiologia e Psichiatria Sociale 11, no. 4: 237–247. 10.1017/S1121189X00005807. 12585014

[eip70055-bib-0033] Merkouris, S. S. , G. Loram , M. Abdelrazek , et al. 2022. “Improving the User Experience of a Gambling Support and Education Website Using a Chatbot.” Universal Access in the Information Society 23, no. 1: 213–225. 10.1007/s10209-022-00932-5.

[eip70055-bib-0034] National Health Service . 2019. “Likemind.” Published Online. https://www.likemind.nhs.uk/.

[eip70055-bib-0035] O'Keeffe, D. , A. Sheridan , A. Kelly , et al. 2016. “A Qualitative Analysis of the Factors That Influence Engagement According to People Diagnosed With a First Episode Psychosis 20 Year Ago.” Early Intervention in Psychiatry 10: 145.

[eip70055-bib-0036] Robson, E. , and K. Greenwood . 2022. “Rates and Predictors of Disengagement and Strength of Engagement for People With a First Episode of Psychosis Using Early Intervention Services: A Systematic Review of Predictors and Meta‐Analysis of Disengagement Rates.” Schizophrenia Bulletin Open 3, no. 1: sgac012. 39144778 10.1093/schizbullopen/sgac012 PMC11205872

[eip70055-bib-0037] Rotondi, A. J. , C. M. Anderson , G. L. Haas , et al. 2010. “Web‐Based Psychoeducational Intervention for Persons With Schizophrenia and Their Supporters: One‐Year Outcomes.” Psychiatric Services 61, no. 11: 1099–1105. 10.1176/ps.2010.61.11.1099. 21041348

[eip70055-bib-0038] Royal College of Psychiatrists . 2022. “National Clinical Audit of Psychosis—England National Report for the Early Intervention in Psychosis 2021/2022.” https://www.hqip.org.uk/national‐programmes.

[eip70055-bib-0039] Sauro, J. 2015. “SUPR‐Q: A Comprehensive Measure of the Quality of the Website User Experience.” Journal of Usability Studies 10, no. 2: 68–86.

[eip70055-bib-0040] Sauro, J. 2018. “10 Things to Know About the SUPR‐Q. Mixed‐Methods Solutions for UX Research.” Accessed June 23, 2023. https://measuringu.com/10‐things‐suprq/.

[eip70055-bib-0041] Tindall, R. , M. Simmons , K. Allott , and B. Hamilton . 2020. “Disengagement Processes Within an Early Intervention Service for First‐ Episode Psychosis: A Longitudinal, Qualitative, Multi‐Perspective Study.” Frontiers in Psychiatry 11: 1–12. 10.3389/fpsyt.2020.00565. 32595545 PMC7304238

[eip70055-bib-0042] Tindall, R. , M. B. Simmons , K. Allott , and B. E. Hamilton . 2018. “Essential Ingredients of Engagement When Working Alongside People After Their First Episode of Psychosis: A Qualitative Meta‐Synthesis.” Early Intervention in Psychiatry 12, no. 5: 784–795. 10.1111/eip.12566. 29624917

[eip70055-bib-0043] Torous, J. , S. Bucci , I. H. Bell , et al. 2021. “The Growing Field of Digital Psychiatry: Current Evidence and the Future of Apps, Social Media, Chatbots, and Virtual Reality.” World Psychiatry 20, no. 3: 318–335. 10.1002/wps.20883. 34505369 PMC8429349

[eip70055-bib-0044] van Deursen, A. , E. Helsper , and R. Eynon . 2016. “Development and Validation of the Internet Skills Scale (ISS).” Information, Communication & Society 19, no. 6: 804–823. 10.1080/1369118X.2015.1078834.

[eip70055-bib-0045] van Deursen, A. , and J. van Dijk . 2011. “Internet Skills and the Digital Divide.” New Media & Society 13, no. 6: 893–911. 10.1177/1461444810386774.

