# Supplementary material for: Implementation of an Online Mental Health Website for the Early Intervention in Psychosis Services, Developed for the Early Youth Engagement (EYE‐2) Trial: A Cross‐Sectional Survey Study of Clinical Barriers and Facilitators to Normalisation
Source: Early Interv Psychiatry. 2025 May 29;19(6):e70055. doi: 10.1111/eip.70055 (PMC12122194; doi:10.1111/eip.70055)
Supplement: Supplementary file 1 — Figure S1. NoMAD scores for ‘sharing feels familiar’. Figure S2. NoMAD scores for ‘feels like current practice’. Figure S3. NoMAD scores for ‘sharing has potential to become routine practice’. Figure S4. Average scores for coherence (individual and collective sense‐making). Figure S5. Average scores for cognitive participation (clinician engagement). Figure S6. Average scores for collective action (the activities involved to make the new practices happen). Figure S7. Average scores for reflexive monitoring (appraisal of the benefits of the intervention). [file EIP-19-0-s001.docx]

Supporting Information

| **Figure SI1 NoMAD scores for 'sharing feels familiar'.**  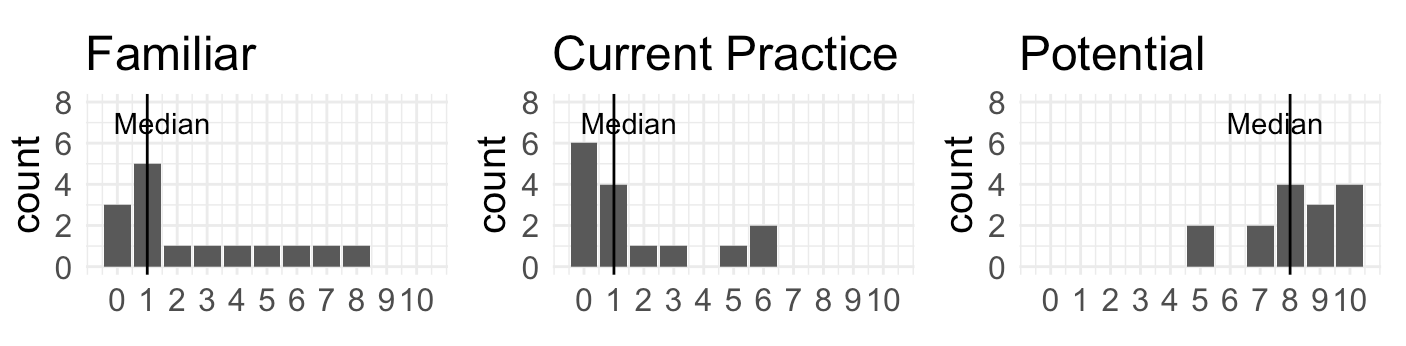 | **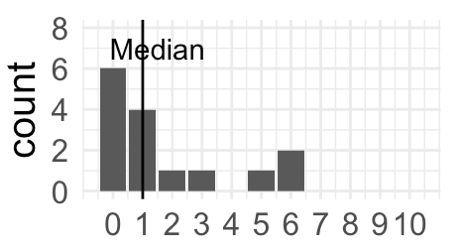Figure SI2 NoMAD scores for 'feels like current practice'.** | **Figure SI3 NoMAD scores for 'sharing has potential to become routine practice'.**  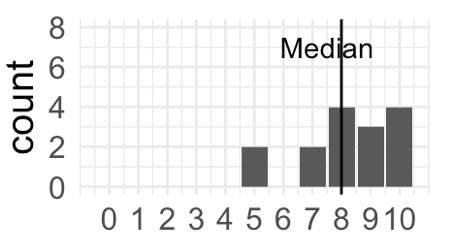 |
| --- | --- | --- |
| A median score was calculated for each participant per construct | | |

| **Figure SI4 Average scores for Coherence (Individual and collective sense making)** | **Figure SI5 Average scores for Cognitive Participation (Clinician engagement)** |
| --- | --- |
| 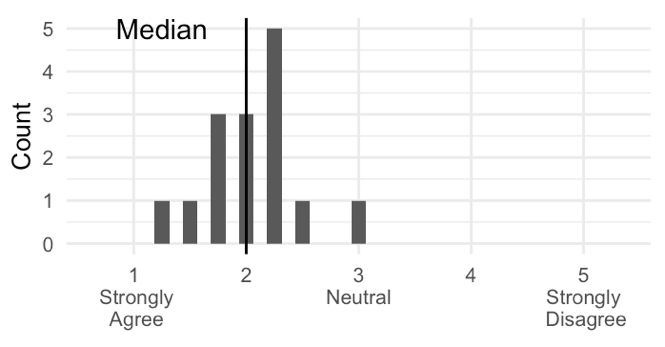 | 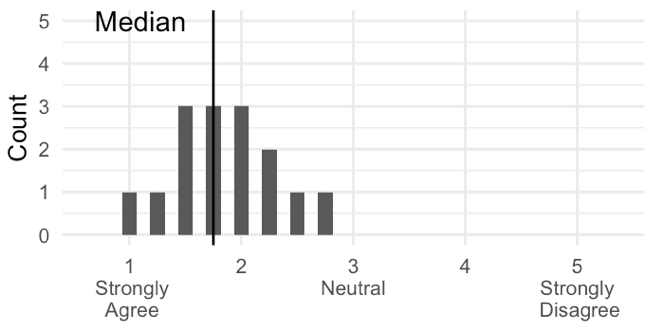 |
| **Figure SI6 Average scores for Collective Action (The activities involved to make the new practices happen)** | **Figure SI7 Average scores for Reflexive Monitoring (Appraisal of the benefits of the intervention)** |
| 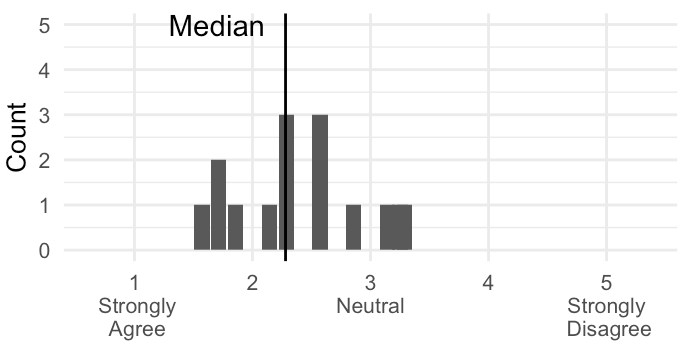 | 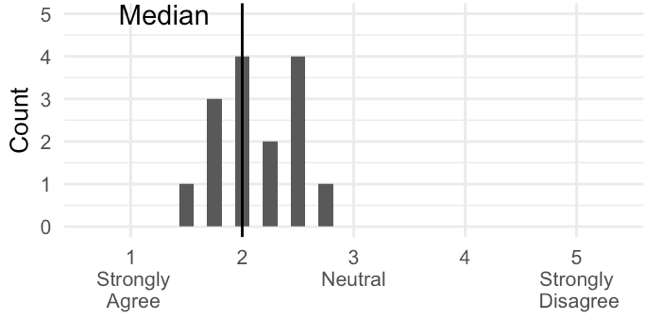 |
| A median score was calculated for each participant per construct | |
